# NiMnO_3_ Anchored on Reduced Graphene Oxide Nanosheets: A New High-Performance Microwave Absorbing Material

**DOI:** 10.3390/nano12071089

**Published:** 2022-03-26

**Authors:** Pin Zhang, Yao Yao, Wenke Zhou, Yawen Liu, Xiaowei Cao, Zhi Zhang

**Affiliations:** 1 Research Center for Camouflage Engineering, The Army Engineering University of PLA, Nanjing 210007, China; zhangpnj01@163.com (P.Z.); liuyawen1111@163.com (Y.L.); 2 State Key Laboratory for Disaster Prevention & Mitigation of Explosion & Impact, The Army Engineering University of PLA, Nanjing 210007, China; yaoylgd@163.com (Y.Y.); zhou.w.k@163.com (W.Z.); 3 Unit 32272 of the People’s Liberation Army, Lanzhou 730030, China

**Keywords:** reduced graphene oxide, NiMnO_3_, microwave absorption, nanocomposite, dielectric loss

## Abstract

With the increasing influence of electromagnetic radiation on precision instruments and organisms, there is an urgent need for research on lightweight and high-strength electromagnetic wave absorbing materials. This study has probed into a new composite absorbing material based on reduced graphene oxide (rGO)-NiMnO_3_, where the like-core-shell NiMnO_3_ is anchored on the rGO nanosheets to significantly improve the electromagnetic wave dissipation ability of the composite material using the inter-component dipole polarization and interface polarization. At the same time, NiMnO_3_ can effectively adjust the impedance matching ratio of rGO so that electromagnetic waves can effectively enter the absorbing material. At a thickness of 3.73 mm, the maximum absorption strength of rGO-NiMnO_3_ reaches −61.4 dB at 6.6 GHz; at a thickness of 2.5 mm, the adequate absorption bandwidth is 10.04–18.00 GHz, achieving a full coverage for the Ku band. As a new option for preparing lightweight and broadband electromagnetic wave absorbing materials, rGO-NiMnO_3_ is an ideal material for electromagnetic wave protection.

## 1. Introduction

Advanced electronic devices are here to stay, but the increasing electromagnetic radiation intensity becomes a stumbling block. Excessive electromagnetic radiation will not only affect the stable operation of precision electronic equipment but also harm the health of organisms [1,2]. Amid the current complex electromagnetic environment, research on high-efficiency, lightweight and broadband electromagnetic wave absorbing materials has become high in the advanced functional material industry [3,4,5].

In recent years, graphene has been widely used in many research fields [6], such as energy materials, photoelectric conversion materials, and superconducting materials, due to their extremely high electrical conductivity and good chemical stability. As an absorbing material, graphene has become a research spotlight due to its high permittivity and low density [7]. However, graphene cannot be simply used as a wave absorber because a large number of electromagnetic waves cannot get into it for dissipation due to its high impedance matching ratio, which is determined by its excessively high conductivity [8]. To improve the impedance matching ratio of graphene without compromising its excellent characteristics such as low density. Graphene composites such as metal sulfides, metal oxides, and magnetic materials are often doped with materials of a low dielectric property to regulate the electromagnetic parameters of the composite to achieve high attenuation loss while ensuring that electromagnetic waves can enter the composite to dissipate. A good example is rGO, such as ZnFe_2_O_3_@SiO_2_@RGO [9], TiO_2_/Ti_3_C_2_T*_x_*/RGO [10], rGO/α-Fe_2_O_3_ [11], and rGO-SCI [12], which has been a hotspot in the electromagnetic wave absorption field. The structural design of the graphene composite will also be taken into consideration. A microstructure can enable multiple reflections of incident electromagnetic waves inside the material to enhance the absorption strength [13,14]. Other common structures include core-shell structures and sandwich-like structures. Zhao et al. have prepared a sandwich microstructured graphene/BaFe_12_O_19_ nanocomposite which could reach up to −40.26 dB with a thickness of 1 mm [15]. This study corroborates that a rationally designed microstructure can significantly increase the absorbing bandwidth of the composite.

As a typical representative binary metal oxide, Ni/Mn-based oxides have certain advantages, good physical and chemical activity, abundant resources, and non-toxicity, so they have been widely studied in many fields. In particular, the perovskite oxide NiMnO_3_ has the advantages of simple synthesis, excellent physical and chemical properties, and low cost, and is widely used in supercapacitors [16], water splitting [17], electrocatalysts [18], and other fields. At the same time, NiMnO_3_ is also an excellent material with low dielectric properties, and it is also one of the preferred materials in the area of electromagnetic absorption [19]. Existing studies are dedicated to combining its inherent low dielectric property with a high dielectric property of other materials to effectively adjust the electromagnetic parameters of composites for a matched impedance, thereby achieving effective absorption of electromagnetic waves [20].

This paper proposes a new strategy: synthesize rGO-NiMnO_3_ by stepwise hydrothermal method. In this study, one-dimensional MnO_2_ nanowires are synthesized first, and then the binary absorbing material rGO-NiMnO_3_ is synthesized. The regulating effect of Ni doping on the absorbing performance of composites is also plumbed. In rGO-NiMnO_3_, NiMnO_3_ forms an electric polarization center, which uses the polarization relaxation mechanism to effectively regulate the impedance matching of the composite, achieving excellent electromagnetic wave absorbing performance and providing a good option for protection against electromagnetic radiation.

## 2. Materials and Methods

### 2.1. Materials

Manganese sulfate monohydrate (MnSO_4_•H_2_O), ammonium persulfate ((NH_4_)S_2_O_8_), nickel chloride hexahydrate (NiCl_2_·6H_2_O), distilled water and absolute ethanol were purchased from Sinopharm Chemical Reagent Co., Ltd. GO was purchased from Nanjing Xianfeng Nanotechnology Co., Ltd. All of the chemical reagents used in this work were analytically pure and were used without further purification.

### 2.2. Preparation of MnO_2_

MnO_2_ nanowires are prepared by the hydrothermal method. First, 1.650 g MnSO_4_•H_2_O is dispersed in 20 mL water for ultrasonic dissolution. Then, 1.826 g (NH_4_)S_2_O_8_ is distributed in 20 mL water for ultrasonic dissolution. Under continuous stirring, the (NH_4_)S_2_O_8_ solution is then slowly pipetted dropwise into the MnSO_4_ solution; the mixed solution is stirred for 20 min. Finally, the hydrothermal reaction is performed at 140 °C for 12 h, and the resulting product is washed several times with distilled water and absolute ethanol and then dried at 50 °C for 12 h.

### 2.3. Preparation of rGO-NiMnO_3_

RGO-NiMnO_3_ is further synthesized by a hydrothermal method based on the MnO_2_ material. First, 100 mg GO is dispersed in a mixed solution of 30 mL water and 30 mL alcohol for one h ultrasonic treatment. Then, 50 mg NiCl_2_·6H_2_O is thoroughly dispersed in 10 mL water and then slowly pipetted dropwise to 20 mL aqueous solution containing MnO_2_ nanowires; the mixed solution is stirred for 20 min. The NiCl_2_-MnO_2_ hybrid solution is pipetted dropwise into the GO solution; the mixed solution is stirred for 30 min before being transferred to a polytetrafluoroethylene liner and placed in a stainless steel reactor (200 mL). The mixture is reacted at 200 °C for 12 h. Finally, the black product is washed with distilled water and ethanol several times and vacuum dried at 50 °C.

### 2.4. Characterization

X-ray diffraction (XRD) was performed using a Bruker D8 Advanced X-ray diffractometer (BRUKER, Karlsruhe, Germany) with Cu K_α_ radiation (λ=1.5406 Å) at 40 KV over the range of 2θ = 5–80°. The scanning electron microscopy (SEM) data of the samples were analyzed using a Hitachi S4800 field emission scanning microscope. The further morphology, crystal structure, and element distribution properties of the rGO-NiMnO_3_ composite were determined by transmission electron microscopy (TEM) with an energy dispersive X-ray spectroscope, which were provided by JEL-2100F (JEOL, Tokyo, Japan). X-ray photon spectroscopy (XPS) was performed on an ESCALAB 250 (Thermo, America).

A vector network analyzer N5224A PNA produced by Agilent Technologies in the United States (Santa Clara, CA) was used to test the electromagnetic parameters of the material. The instrument has a maximum output frequency of 43.5 GHz, an output power of 13 dBm, built-in 2 or 4 ports, and can scan 201 data points at a time. The absorbing material and paraffin are mixed uniformly according to a certain proportion to prepare a coaxial ring with an outer diameter of 7 mm and an inner diameter of 3.04 mm. Based on the coaxial probe method, the real (*ε*′ and *µ*′) and imaginary (*ε*″ and *μ*″) parts of permittivity and permeability of the sample in the range of 2–18 GHz were obtained by putting the coaxial ring in the vector network analyzer to texting. After that, the reflection loss of the samples was calculated from the measured electromagnetic parameters using the transmission line theory, which was based on infinite transverse dimensions. The calculated reflection loss is generally considered to be the absorption performance of the material under the normal incidence of electromagnetic waves. Further analysis on the angle of the incidence electromagnetic waves is in the supporting information. A schematic diagram of the electromagnetic wave absorption process is shown in Figure S1. When the incident electromagnetic wave reaches the surface of the absorber, a part of the electromagnetic wave will be emitted from the surface, and the rest of the electromagnetic wave will enter the inside of the absorber. Then, the absorbing material uses multiple loss mechanisms to convert the electrical and magnetic energy carried by the electromagnetic wave into heat energy and dissipate it to achieve the electromagnetic wave absorption effect.

## 3. Results

**Figure 1:**
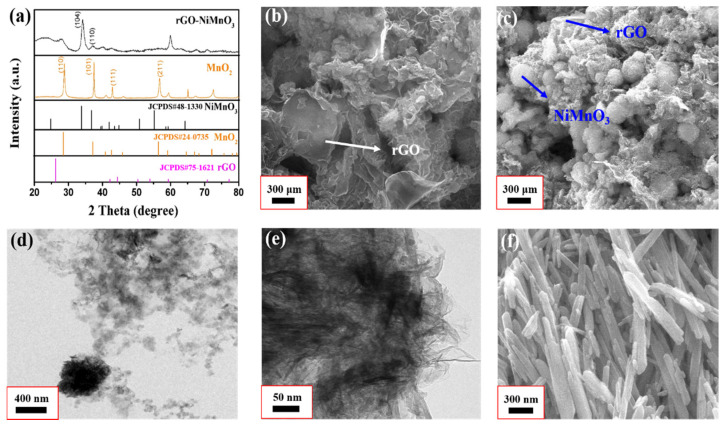
(**a**) XRD patterns of rGO-NiMnO_3_ and MnO_2_ nanowires; (**b**,**c**) SEM images of rGO-NiMnO_3_; (**d**,**e**) TEM images of rGO-NiMnO_3_; (**f**) SEM images of MnO_2_ nanowires.

To verify the accuracy of the prepared materials, all of the samples are analyzed by X-ray diffraction analysis technology. The test results are shown in Figure 1a. Figure 1a shows the XRD patterns of rGO-NiMnO_3_ and MnO_2_ in the range 2*θ* = 20~80°, respectively. From the XRD pattern of MnO_2_, the characteristic diffraction peaks at 2*θ* = 28.4°, 37.2°, 42.5°, and 56.7° correspond to (110), (101), (111), and (211) crystal planes in the MnO_2_ standard pattern (JCPDS#24-0735), respectively, indicating that the MnO_2_ material with good crystal phase is successfully prepared by the hydrothermal method. For rGO-NiMnO_3_, the diffraction peaks at 2*θ* = 33.961° and 36.739° match the (104) and (110) crystal planes in the standard NiMnO_3_ pattern (JCPDS#48-1330). The apparent deviation of the diffraction peak at 2*θ* = 59.848° relative to that of NiMnO_3_ may result from partial defects in the crystal lattice caused by the uneven Ni distribution during the preparation of NiMnO_3_. Since rGO has a much lower crystallinity than NiMnO_3_, its diffraction peak at 2*θ* = 26.228° cannot be clearly seen in the XRD pattern of rGO-NiMnO_3_.

Figure 1b,c is SEM images of rGO-NiMnO_3_ at different scales. From Figure 1c, NiMnO_3_ is spherically distributed on the rGO nanosheet, and the NiMnO_3_ nanospheres are uniform in size, about 2 µm, with wrinkles on the surface. A like-core-shell structure is formed, where Ni–O compounds are mainly distributed outside the core-shell structure, and Mn–O compounds are mainly distributed inside the core-shell structure. The Mn–O compound comprises the electric polarization center, while the Ni–O compound contacts with the Mn–O compound and rGO to form the Mn–Ni and Ni–C interfaces, resulting in interface polarization, which effectively regulates the impedance matching of the composites, thus achieving a good absorption of incident electromagnetic waves. Figure 1d,e is the TEM image of rGO-NiMnO_3_ at different magnification. It can be seen from Figure 1d that NiMnO_3_ nanospheres are embedded on the rGO nanosheets. The wrinkles on the surface of the NiMnO_3_ nanospheres can be clearly seen in Figure 1e. Figure 1f shows the MnO_2_ precursor material prepared by the hydrothermal method. This material is distributed as one-dimensional nanowires, with a smooth surface but different scales and low chemical stability.

**Figure 2:**
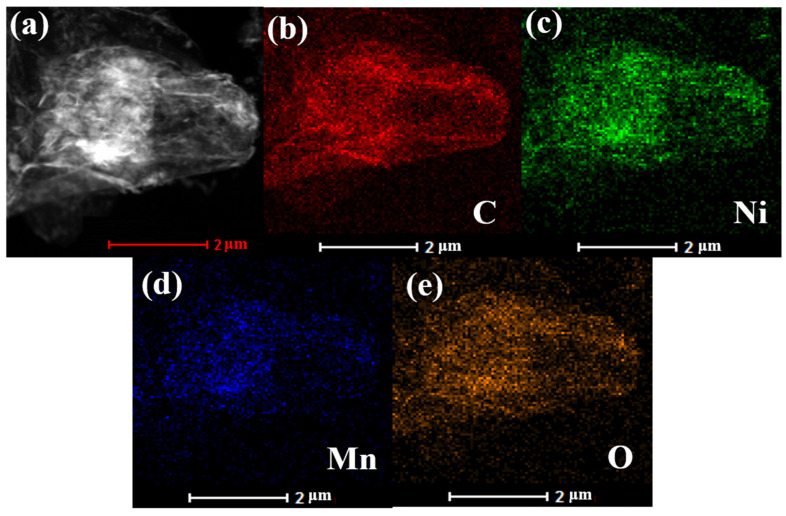
TEM Mapping diagram of rGO-NiMnO_3_. (**a**) TEM diagram of rGO-NiMnO_3_; (**b**) The distribution of C elements; (**c**) The distribution of Ni elements; (**d**) The distribution of Mn elements; (**e**) The distribution of O elements.

To further verify the completeness of rGO-NiMnO_3_, the surface element distribution analysis of the material is carried out. From Figure 2, the four elements of C, Ni, Mn, and O are present in the rGO-NiMnO_3_, and C is relatively densely distributed, showing that rGO nanosheets, as a substrate material, are anchored with NiMnO_3_. Therefore, the rGO-NiMnO_3_ composite is successfully prepared by the stepwise hydrothermal method.

**Figure 3:**
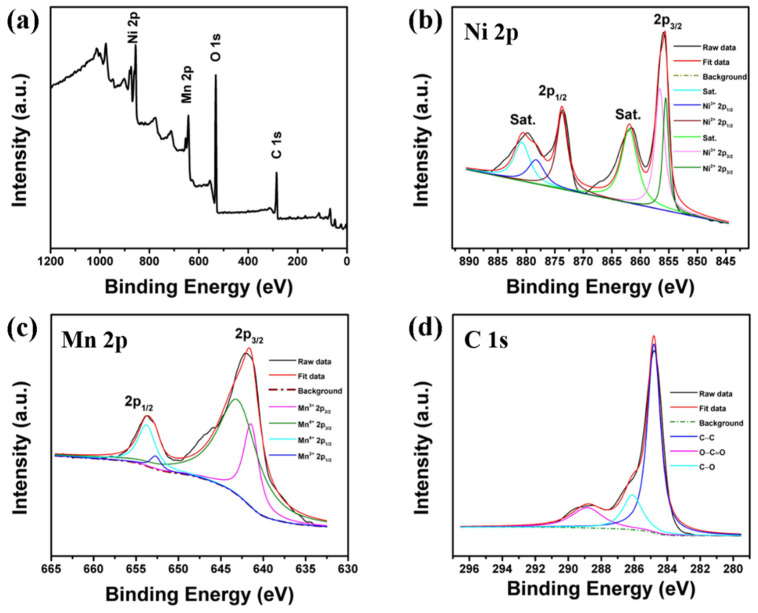
XPS spectra of the rGO-NiMnO_3_ (**a**) survey spectrum, (**b**) Ni 2p, (**c**) Mn 2p, and (**d**) C 1s.

X-ray photoelectron spectroscopy (XPS) is explored to determine the surface chemical states of rGO-NiMnO_3_. Figure 3a shows the survey spectrum of the synthesized sample consisting of C 1s, O 1s, Mn 2p, and Ni 2p peaks. The Ni 2p spectrum of rGO-NiMnO_3_ (Figure 3b) exhibits two spin–orbit doublets, which can be ascribed to Ni^2+^, Ni^3+^. The Ni^2+^ components are located at binding energies of 855.5 and 873.7 eV; the Ni^3+^ features are centered at 856.4 and 878.2 eV. Besides, the separate peaks sited at 861.9 and 880.7 eV are satellites. For the Mn 2p spectrum of rGO-NiMnO_3_ in Figure 3b, two spin–orbit doublets can be observed. The peaks at the binding energy of 643 and 653.75 eV can correspond to Mn^4+^. The peaks located at 641.3 and 652.5eV can be complementary to Mn^3+^ [21]. The C 1s spectrum (Figure 3d) of rGO-NiMnO_3_ exhibits three components at 288.8, 286.1, 284.6 eV, which can be indexed to the O=C–O, C–O, and C–C, respectively. According to the XPS results, the rGO-NiMnO_3_ has been successfully synthesized.

## 4. Discussion

**Figure 4:**
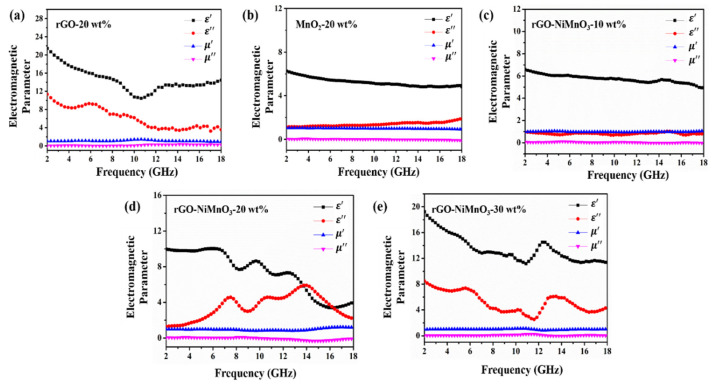
Electromagnetic parameters of rGO, MnO_2_, and rGO-NiMnO_3_, (**a**) rGO-20 wt%, (**b**) MnO_2_-20 wt%, (**c**) rGO-NiMnO_3_-10 wt%, (**d**) rGO-NiMnO_3_-20 wt%, and (**e**) rGO-NiMnO_3_-30 wt%.

The absorption strength of electromagnetic wave absorbing materials is usually measured in two ways. One is to use the coaxial ring method to obtain the electromagnetic parameters of the absorbing material. Through the analysis of the electromagnetic parameters, the value of the reflection loss of the absorbing material is obtained by numerical simulation [22,23]. Electromagnetic parameters are the primary basis for studying the properties of absorbing materials [24]. There are four parameters: real part ($\varepsilon^{\prime }$) and imaginary part ($\varepsilon^{\prime \prime }$) of permittivity, real part ($\mu^{\prime }$) and imaginary part ($\mu^{\prime \prime }$) of permeability [25]. The real parts of permittivity and permeability reflect the capacity of the absorbing material to store the electric and magnetic energy carried by incident electromagnetic waves, while the imaginary parts of permittivity and permeability reflect the ability of the absorbing material to dissipate the electric and magnetic energy carried by incident electromagnetic waves [26]. The other is to use the arch method to measure the reflection loss of the absorber directly. In this study, the first method is used to obtain the electromagnetic parameters of rGO, MnO_2_, and rGO-NiMnO_3_ at different doping levels. The results are shown in Figure 4. Figure 4a,b respectively show the electromagnetic parameters of rGO and MnO_2_ when the doping level is 20 wt%, and both materials exhibit dielectric property but no magnetic loss property. rGO has a higher complex permittivity, which is related to its extremely high electrical conductivity. MnO_2_ has a lower complex permittivity due to its lower conductivity. Figure 4c–e shows the electromagnetic parameters of rGO-NiMnO_3_ with doping levels of 10 wt%, 20 wt%, and 30 wt%, respectively. As the doping level increases, the complex permittivity of the composite also increases to a certain extent. The complex permittivity value of rGO-NiMnO_3_-20 wt% fluctuates greatly compared with the other two doping levels, and it shows noticeable vibration, especially around 8.0 GHz, 10 GHz, and 14 GHz. For dielectric loss absorbing materials, fluctuations in the real and imaginary parts of the permittivity often bring about changes in the electromagnetic wave absorption performance, and might lead to the appearance of absorption peaks [27,28]. Therefore, it can be inferred that some absorption peaks may appear around 8.0 GHz, 10 GHz, and 14 GHz under the doping level of 20 wt%. 

**Figure 5:**
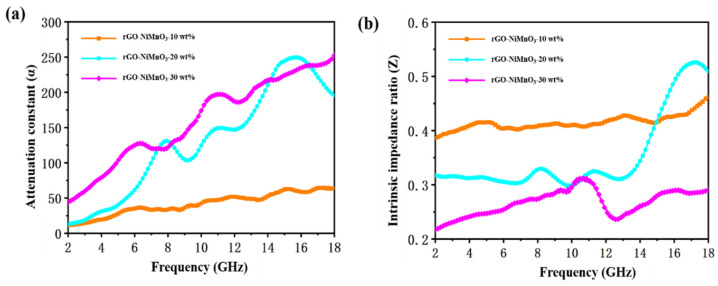
Attenuation constant (**a**) and impedance matching ratio (**b**) of rGO-NiMnO_3_ with different doping levels.

To further study the influence of doping level on the electromagnetic wave absorbing performance of rGO-NiMnO_3_, the attenuation loss (*α*) and intrinsic impedance ratio (*Z*) of the composites under three doping levels are studied. In terms of transmission line theory, the relationship between α and the incident electromagnetic wave frequency can be expressed by Equation (1) [29]:

(1) $$\alpha =\frac{\sqrt{2}\pi f}{c}\times \sqrt{\left(\mu^{\prime \prime }\varepsilon^{\prime \prime }-\mu^{\prime }\varepsilon^{\prime }\right)+\sqrt{{\left(\mu^{\prime \prime }\varepsilon^{\prime \prime }-\mu^{\prime }\varepsilon^{\prime }\right)}^{2}+{\left(\mu^{\prime }\varepsilon^{\prime \prime }+\mu^{\prime \prime }\varepsilon^{\prime }\right)}^{2}}}$$

where f represents the frequency of incident electromagnetic waves, and c represents the speed of light in a vacuum. The intrinsic impedance ratio can be calculated as follows [30]:

(2) $$Z=Z_{r}/Z_{0}$$

(3) $$Z_{r}=Z_{0}\sqrt{\mu_{r}/\varepsilon_{r}}$$

(4) $$\varepsilon_{r}=\varepsilon^{\prime }+j\varepsilon^{\prime \prime }$$

(5) $$\mu_{r}=\mu^{\prime }+j\mu^{\prime \prime }$$

where $Z_{r}$ is the intrinsic impedance of absorber, $Z_{0}$ is the free space impedance, $\varepsilon_{r}$ is complex permittivity, and $\mu_{r}$ is complex permeability. In Figure S2, the attenuation loss and intrinsic impedance ratio of rGO and MnO_2_ have been presented. It can be seen that the α value of rGO is over 100 from 4 to 18 GHz, and the α value of MnO_2_ is below 100. In contrast, the *Z* value of MnO_2_ is overall higher than that of rGO. All of the *Z* value of MnO_2_ is over 0.4, while the *Z* value of rGO is over 0.3 only from 9 to 12 GHz. Usually, when the attenuation constant is higher than 100, it is considered that the absorber can effectively lose the incident electromagnetic wave. When the intrinsic impedance ratio is higher than 0.3, the electromagnetic wave can enter the absorber in large quantities without being reflected by the surface [31,32]. The low intrinsic impedance ratio of rGO (Z<0.3) indicates that a large number of electromagnetic waves are reflected on the surface of rGO and cannot enter the interior. Therefore, although rGO has a strong attenuation ability to incident electromagnetic waves, its actual wave absorption performance is weak. MnO_2_ shows the exact opposite properties to rGO, in which electromagnetic waves can be incident in a large amount, but no effective loss can be obtained. Therefore, the combination of MnO_2_ and rGO can effectively adjust the intrinsic impedance ratio and attenuation loss characteristics of the composite, and it is expected to achieve good electromagnetic wave absorption performance. Figure 5 shows the α and *Z* results of rGO-NiMnO_3_-10 wt%, rGO-NiMnO_3_-20 wt%, and rGO-NiMnO_3_-30 wt%. According to Figure 5, rGO-NiMnO_3_-10 wt% has the best impedance matching ratio but lower attenuation loss ability, showing that electromagnetic waves can enter the wave absorber under this doping level but can hardly be absorbed due to insufficiency of absorber content. rGO-NiMnO_3_-30 wt% has the strongest attenuation loss capability but lower impedance matching ratio, showing that the increased absorber content has significantly improved the attenuation loss ability of incident electromagnetic waves under this doping level due to, but hindered electromagnetic waves from entering the absorber, as most electromagnetic waves have bounced off the surface of the absorber. rGO-NiMnO_3_-20 wt% has a moderate impedance matching ratio and attenuation loss ability, and therefore it can be inferred that the composite exhibits the highest electromagnetic wave absorption strength under this doping level. It is also worth noting that rGO-NiMnO_3_-20 wt% has a very high attenuation loss value and impedance matching ratio (close to 1) in the 14.0–18.0 GHz frequency band, showing that electromagnetic waves in this frequency band can not only enter the rGO-NiMnO_3_-20 wt% material in large quantities but also achieve effective attenuation. The effective electromagnetic wave absorption bandwidth of rGO-NiMnO_3_-20 wt% is expected to cover 14.0–18.0 GHz.

**Figure 6:**
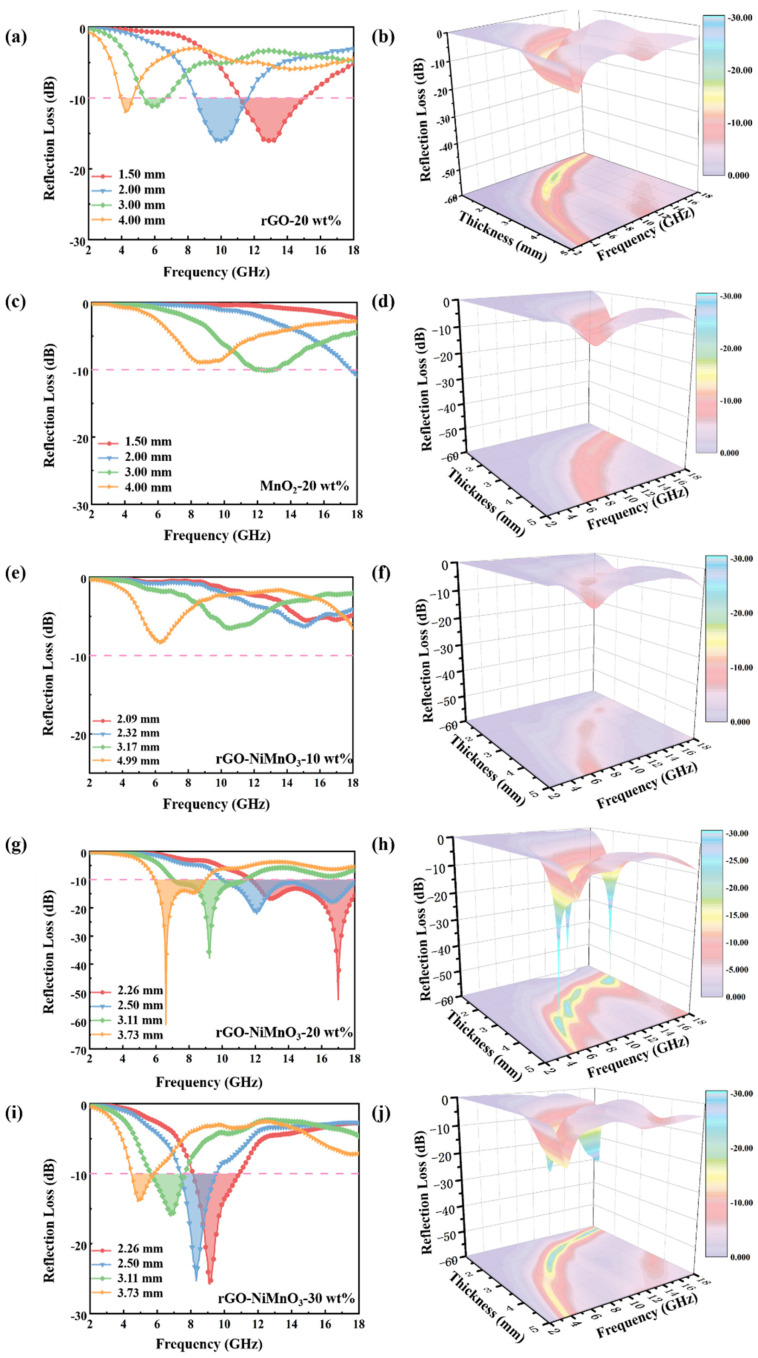
The 2D and 3D reflection loss diagram of rGO, MnO_2_ and rGO-NiMnO_3_ in 2-18 GHz. (**a**,**b**) rGO-20 wt%; (**c**,**d**) MnO_2_-20 wt%; (**e**,**f**) rGO-NiMnO_3_-10 wt%; (**g**,**h**) rGO-NiMnO_3_-20 wt%; (**i**,**j**) rGO-NiMnO_3_-30 wt%.

To further study the electromagnetic wave absorption strength of rGO, MnO_2,_ and rGO-NiMnO_3_ composites in the range of 2–18 GHz, the electromagnetic parameters of the three materials in Figure 4 are used to calculate their respective reflection loss values, which can be calculated as follows [10,33]:

(6) $$Z_{in}=Z_{0}\sqrt{\frac{\mu_{r}}{\varepsilon_{r}}}\tanh\left(j\frac{2\pi fd}{c}\sqrt{\mu_{r}\varepsilon_{r}}\right)$$

(7) $$RL\left(dB\right)=20\mathrm{lg}\left|\frac{Z_{in}-Z_{0}}{Z_{in}+Z_{0}}\right|$$

where $Z_{in}$ is the input impedance, $Z_{0}$ is the free space impedance, f is the frequency of the incident electromagnetic wave, c  is the speed of light in vacuum, d is the thickness of the absorbing material, and h  is the Planck constant. The frequency range where the reflection loss value is less than −10 dB is called the effective absorption bandwidth of the absorbing material. Figure 6a,c respectively show the reflection loss values (2D) of rGO and MnO_2_ at four typical absorber layer thicknesses (1.50 mm, 2.00 mm, 3.00 mm, and 4.00 mm), and Figure 5b,d respectively show the reflection loss values (3D) of rGO and MnO_2_ with an absorber layer thickness of 1–5 mm. From the figure, rGO exhibits a certain absorption strength due to its high complex permittivity, but the maximum absorption strength in the thickness range of 1–5 mm is less than −15 dB due to the low impedance matching. The strength needs to be improved. For MnO_2_, the maximum absorption strength cannot reach −10 dB in the thickness range of 1–5 mm, and there is no effective absorption frequency band for electromagnetic waves in 2–18 GHz; namely, the single MnO_2_ has no pronounced absorbing effect. Figure 6e–j corresponds to the reflection loss diagram (2D) and the reflection loss map (3D) of rGO-NiMnO_3_ under the typical thickness and the thickness range of 1–5 mm with 10 wt%, 20 wt%, and 30 wt% doping levels, respectively. From the figure, rGO-NiMnO_3_-10 wt% cannot achieve effective absorption of electromagnetic waves at any thickness (1–5 mm), while rGO-NiMnO_3_-30 wt% exhibits a specific absorption strength. For rGO-NiMnO_3_-30 wt%, the best absorber thickness is 2.26 mm, 2.50 mm, 3.11 mm, and 3.73 mm. As the absorber thickness increases, the absorption strength decreases. At 30 wt% doping, the composite achieves the highest absorption strength of −25.8 dB at 9.12 GHz, and the corresponding effective absorbing bandwidth is 2.72 GHz (8.16–10.88 GHz). The strength needs to be improved. Compared with the other two doping levels, rGO-NiMnO_3_-20 wt% exhibits the highest electromagnetic wave absorption strength, which can be proved from Figure 6g,h. Figure 6g shows the reflection loss values of rGO-NiMnO_3_-20 wt% at four typical thicknesses. When the thickness is 2.26 mm and 3.73 mm, the composite has higher electromagnetic wave absorption strength. 

**Figure 7:**
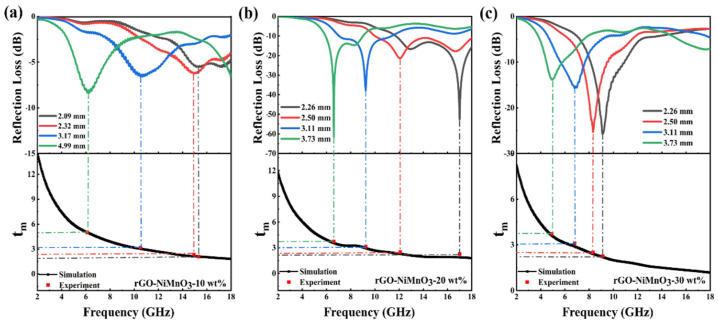
Comparison of various absorber thickness (*t_m_*) for (**a**) rGO-NiMnO_3_-10 wt%, (**b**) rGO-NiMnO_3_-20 wt%, and (**c**) rGO-NiMnO_3_-30 wt% with the simulated thickness under λ/4 conditions at the frequency of maximum reflection loss values ($f_{m}$).

The absorption strength reaches −51.5 dB and −62.4 dB, respectively. It is worth noting that at the thickness of 2.26 mm, the composite exhibits a high electromagnetic wave absorption strength, and the corresponding effective absorption bandwidth reaches 6.24 GHz (11.76–18.00 GHz). From a practical point of view, the effective absorbing bandwidth of rGO-NiMnO_3_-20 wt% at a thickness of 2.5 mm can reach 7.96 GHz (10.04–18.00 GHz). The related microwave absorbers of carbon-based nanocomposites are shown in Table 1. This strength not only completely covers the Ku frequency band but also partially covers the X frequency band, demonstrating an extremely high practical value. The high electromagnetic wave absorption strength of rGO-NiMnO_3_ can be attributed to the effective regulation of the impedance matching of rGO by NiMnO_3_. As an electric polarization center in the composite, NiMnO_3_ uses dipole polarization to consume electromagnetic waves significantly and fully converts the electric energy in incident electromagnetic waves into thermal energy for dissipation. The Ni-Mn interface and Ni-C interface produce interface polarization under the action of electromagnetic waves, which also helps dissipate incident electromagnetic waves. Otherwise, it can be seen that with the increase of the thickness of the absorber, the absorption peak gradually moves to the low frequency, which can be explained by the quarter-wavelength theory. The thickness of the absorber (*t_m_*) at the peak frequency ($f_{m}$) can be calculated as follows [5]:

(8) $$t_{m}=\frac{n\lambda }{4}=\frac{nc}{\left(4f_{m}\sqrt{\left|\mu_{r}\right|\left|\varepsilon_{r}\right|}\right)},\left(n=1,3,5,\ldots \right)$$

where λ is the wavelength of the incident electromagnetic wave. The relationship between the thickness of the absorbers and the simulated thickness under λ/4 conditions at the frequency of maximum reflection loss values is shown in Figure 7 and Figure S3. The red squares represent the specific thickness of the strongest absorption peaks, which are all near the λ/4 curve. This indicates that the surface reflected wave and the second reflected wave on the bottom of the material could produce an interference cancellation effect, which is conducive to weakening the reflected intensity of electromagnetic waves. 

## 5. Conclusions

In summary, rGO nanosheets anchored with NiMnO_3_, a composite with significant electromagnetic wave absorbing performance, are successfully prepared by a stepwise hydrothermal method. The results show that NiMnO_3_ is successfully anchored on the rGO nanosheets. Compared with the two precursor materials, rGO and MnO_2_, the electromagnetic wave absorption strength of rGO-NiMnO_3_ is significantly enhanced due to the improved impedance matching and dielectric loss ability. The dielectric loss of the composite can be attributed to the dipole polarization relaxation, interface polarization, and the unique microstructure that promotes electron transport in nanocomposites. rGO nanosheets anchored with NiMnO_3_ are arguably a good choice as an electromagnetic wave absorber, and they provide a new perspective for the application of wide bandgap semiconductor materials in the electromagnetic field.

## Figures and Tables

**Table 1:** Microwave absorption properties of carbon-based nanocomposites.

Samples	Mass Ratio (%)	Thickness (mm)	Frequency Range (GHz) (RL ≤ −10 dB)	RL (min) (dB)	Ref.
TiO_2_/Ti_3_C_2_T*_x_*/RGO	10	2.5	~9.20–12.20 (3.0)	−65.3	[10]
BaFe_12_O_19_/RGO	15	1	13.13–17.00 (3.87)	−40.26	[15]
PPy/carbon microspheres	40	3	11.17–12.26 (1.09)	−38.1	[29]
Co_0.5_Cu_0.5_Fe_2_O_4_/RGO	unavailable	3.17	~5.20–7.80 (2.6)	−58.25	[33]
rGO-NiMnO_3_	20	2.5	10.04–18.00 (7.96)	−21.4	This work

## Data Availability

Not applicable.

## Supplementary Materials

The following supporting information can be downloaded at: https://www.mdpi.com/article/10.3390/nano12071089/s1, Figure S1: The process of electromagnetic wave absorption; Figure S2. Attenuation constant (a) and impedance matching ratio (b) of rGO and MnO_2_; Figure S3: Comparison of various absorber thickness (*t_m_*) for (a) MnO_2_-20 wt% and (b) rGO-20 wt% with the simulated thickness under λ/4 conditions at the frequency of maximum RL values ($f_{m}$).
