# Supplementary material for: NiMnO3 Anchored on Reduced Graphene Oxide Nanosheets: A New High-Performance Microwave Absorbing Material
Source: Nanomaterials (Basel). 2022 Mar 26;12(7):1089. doi: 10.3390/nano12071089 (PMC9000542; doi:10.3390/nano12071089)
Supplement: Supplementary file 1 [file nanomaterials-12-01089-s001.zip › nanomaterials-1648089-supplementary.pdf]

Supporting Information

$NiMnO_3$ Anchored on Reduced Graphene Oxide Nanosheets: A New High-Performance Microwave Absorbing Material

Pin Zhang [1], Yao Yao [2], Wenke Zhou [2], Yawen Liu [1], Xiaowei Cao [3,*] and Zhi Zhang [2,*]

[1] Research Center for Camouflage Engineering, The Army Engineering University of PLA, Nanjing 210007, China; zhangpnj01@163.com (P.Z.); liuyawen1111@163.com (Y.L.)
[2] State Key Laboratory for Disaster Prevention & Mitigation of Explosion & Impact, The Army Engineering University of PLA, Nanjing 210007, China; yaoylgd@163.com (Y.Y.); zhou.w.k@163.com (W.Z.)
[3] Unit 32272 of the People's Liberation Army, Lanzhou 730030, China
* Correspondence: caoxiaowei2012@126.com (X.C.); zhangnjjn@163.com (Z.Z.)

2.4. Characterization

In fact, the reflection loss calculated using transmission line theory is generally considered to be the material's absorbing properties under the condition of normal incidence of electromagnetic waves. Because the electromagnetic wave has the slightest contact with the absorber when the electromagnetic wave is vertically incident, the reflection loss performance of the absorber obtained in this case is much lower than that of the electromagnetic wave obliquely incident. If the absorbing material exhibits excellent electromagnetic wave absorption performance when the electromagnetic wave is incident vertically, then the electromagnetic wave absorption performance of the absorbing material will be more excellent when the electromagnetic wave is incident obliquely. Therefore, the absorbing properties of the samples under the vertical incidence of electromagnetic waves have been studied in the manuscript, while the case of oblique incidence of electromagnetic waves was not discussed.

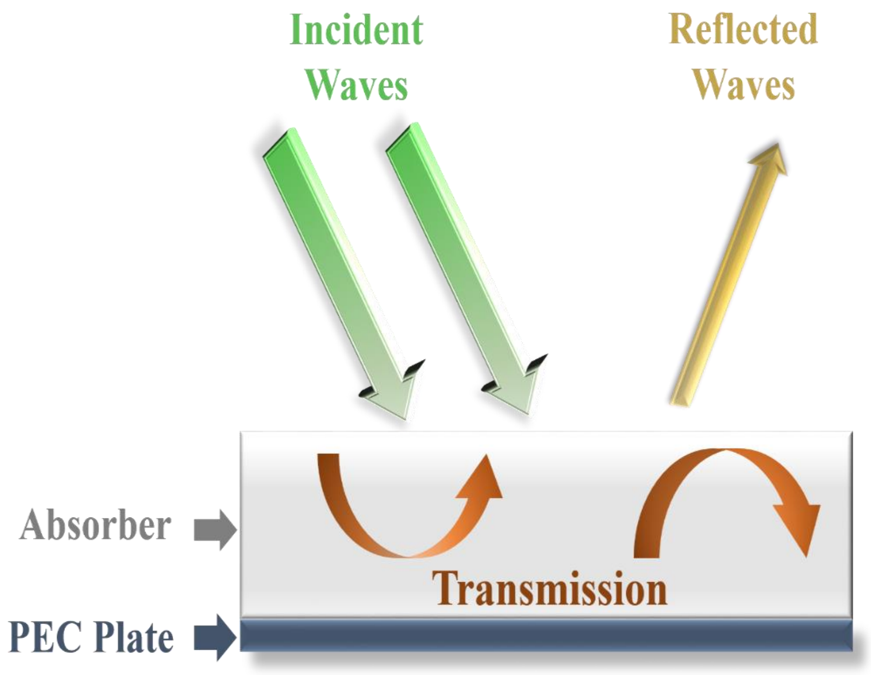

Figure S1. The process of electromagnetic wave absorption.

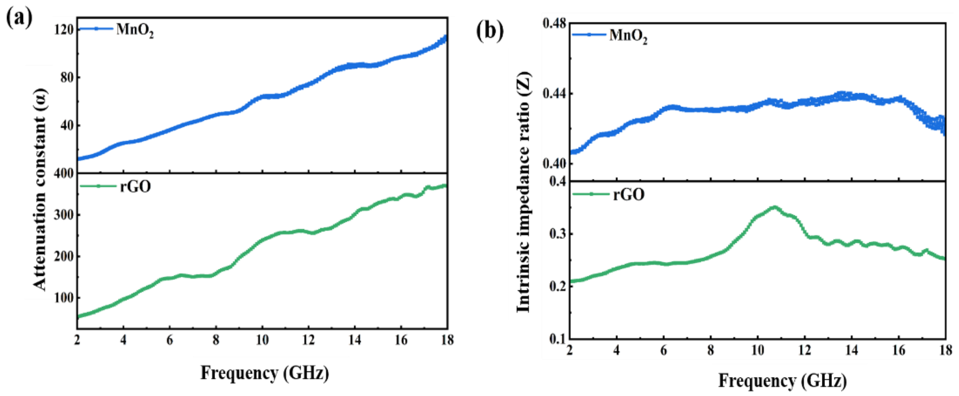

Figure S2. Attenuation constant (a) and impedance matching ratio (b) of rGO and MnO_2.

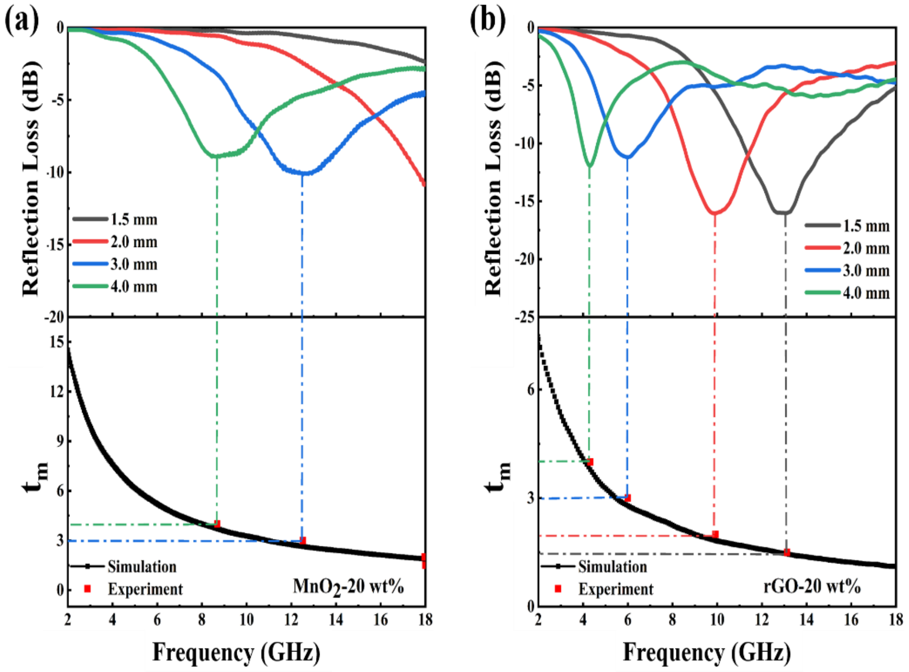

Figure S3. Comparison of various absorber thickness (t_m) for (a) MnO_2-20 wt% and (b) rGO-20 wt% with the simulated thickness under $\lambda/4$ conditions at the frequency of maximum RL values (f_m).
